# Predictability of Intraocular Lens Power Calculation for Cataract with Keratoconus: A Multicenter Study

**DOI:** 10.1038/s41598-018-20040-w

**Published:** 2018-01-22

**Authors:** Kazutaka Kamiya, Kei Iijima, Shoji Nobuyuki, Yosai Mori, Kazunori Miyata, Takefumi Yamaguchi, Jun Shimazaki, Shinya Watanabe, Naoyuki Maeda

**Affiliations:** 1 Department of Ophthalmology, Kitasato University, Kanagawa, Japan; 2 Department of Ophthalmology, Miyata Eye Hospital, Miyazaki, Japan; 3 Department of Ophthalmology, Tokyo Dental College Ichikawa Hospital, Chiba, Japan; 4 Department of Ophthalmology, Osaka University Graduate School of Medicine, Osaka, Japan; 5 Present Address: School of Allied Health Sciences, Kitasato University, Kanagawa, Japan

## Abstract

This study was aimed to assess the predictability of intraocular lens (IOL) power calculation after cataract surgery for keratoconus. We retrospectively reviewed the clinical charts of 102 eyes of 71 consecutive keratoconic patients who developed cataract. We determined manifest spherical equivalent, prediction errors, and absolute errors, 1 month postoperatively. The achieved refraction was significantly more hyperopic than the targeted refraction, when keratometric readings were used (p = 0.001). At 1 month, 36% and 63% of the eyes were within ±0.5 and ±1.0 D, respectively, of the targeted correction. We found a significant correlation between the prediction error and the mean keratometry (Pearson correlation coefficient r =−0.545, p < 0.001). No vision-threatening complications occurred in any case. The achieved refraction was significantly more myopic than the targeted refraction, when total corneal refractive power was used (p = 0.013). Phacoemulsification with IOL implantation appeared to be safe and effective, and the accuracy was also good in mild keratoconus, but not in severe keratoconus. It should be noted that that a large amount of hyperopic shift occurred especially in advanced keratoconic patients, when keratometric readings were used for IOL power calculation, and that a slight, but significant, myopic shift occurred, when total corneal refractive power was used.

## Introduction

Keratoconus is a progressive disorder characterized by ectasia and thinning of the cornea. The progressive thinning and subsequent anterior protrusion of the cornea can result in not only severe myopic astigmatism but also asymmetrical irregular astigmatism, leading to distorted vision. Considering that keratoconic eyes tends to develop cataract earlier than non-keratoconus patients^1,2^, it is reasonable that the number of patients requiring cataract surgery has been increased with aging. It is still challenging for the precise intraocular lens (IOL) power calculation for such patients in daily practice, since it is sometimes difficult to accurately determine the keratometric readings, especially in keratoconic eyes having skewed hemi-meridians. Moreover, the keratometric readings may differ from the total corneal refractive power especially in keratoconic patients, because the former readings are theoretically calculated based on the assumption that the ratio of the anterior and posterior curvatures was constant as normal eyes. Accurate IOL power calculation is mandatory for better visual and refractive outcomes and subsequent patient satisfaction, even in keratoconic patients having cataract. There have been so far several studies on the clinical outcomes of non-toric IOL implantation^1–3^ and those of toric IOL implantation^4–10^ for keratoconus. However, most studies were performed in a single center with a small sample size, and were merely focused on the surgical outcomes of cataract surgery only using one IOL power calculation formula. Accordingly, the predictability of IOL power calculation using the keratometric readings as well as the total corneal refractive power or using several IOL calculation formulas in a large cohort of keratoconic patients has not been elucidated so far. It may give intrinsic insights on the further improvement of the predictability of cataract surgery in these patients in the future. The purpose of the current study is to retrospectively assess the clinical outcomes of cataract surgery in a large cohort of keratoconic patients presenting at major clinical centers in Japan, with special attention to the refractive accuracy of IOL power calculation.

## Results

### Patient Demographics

Preoperative and postoperative patient demographics are listed in Table 1. The patient age at the time of surgery was 61.0 (53.0, 67.8) (median (25th and 75th percentile)) years. All surgeries were uneventful and no intraoperative complication was observed in this series.

**Table 1:** Preoperative demographics of the study population in eyes undergoing intraocular lens implantation for keratoconus.

Preoperative demographics (median (25th and 75th percentile))	
Number of eyes	101
Male:Female	32:38
Age	61.0 (53.0, 68.0) years
LogMAR UDVA	1.30 (0.82, 1.70)
LogMAR CDVA	0.30 (0.15, 0.52)
Manifest sphere (D)	−5.50 (−10.00, −0.50)
Manifest cylinder (D)	−2.50 (−3.63, −1.00)
Astigmatic axis (degree)	80 (35, 130)
Mean keratometric readings	46.6 (45.1, 48.9) D
Axial length	25.81 (24.62, 27.25) mm
Amsler-Krumeich classification	Grade 1 (65 eyes), Grade 2 (20 eyes), Grade 3 (8 eyes), and Grade 4 (8 eyes)
Postoperative demographics (median (25th and 75th percentile))	
LogMAR UDVA	0.35 (0.15, 0.70)
LogMAR CDVA	0.00 (−0.08, 0.10)
Manifest sphere (D)	−0.50 (−1.75, 0.25)
Manifest cylinder (D)	−1.50 (−3.00, −0.50)
Astigmatic axis (degree)	90 (30, 125)

logMAR = logarithm of the minimal angle of resolution, UDVA = uncorrected distance visual acuity, CDVA = corrected distance visual acuity, D = diopter.

### Safety and Efficacy

LogMAR UDVA was significantly improved from 1.30 (0.82, 1.70) preoperatively to 0.35 (0.15, 0.70) postoperatively (p < 0.001, Student t test). LogMAR CDVA was also significantly improved from 0.30 (0.15, 0.52) preoperatively to 0.00 (−0.08, 0.10) postoperatively (p < 0.001). The manifest spherical equivalent was significantly changed from -7.00 (−11.00, −2.38) D preoperatively to −1.75 (−2.75, −0.50) D postoperatively (p < 0.001). Of the 27 eyes targeted for emmetropia, 22 (81%) and 10 (37%) achieved UDVAs of 20/40 and 20/20 or better at 1 month, respectively.

### Predictability

**Figure 1:**
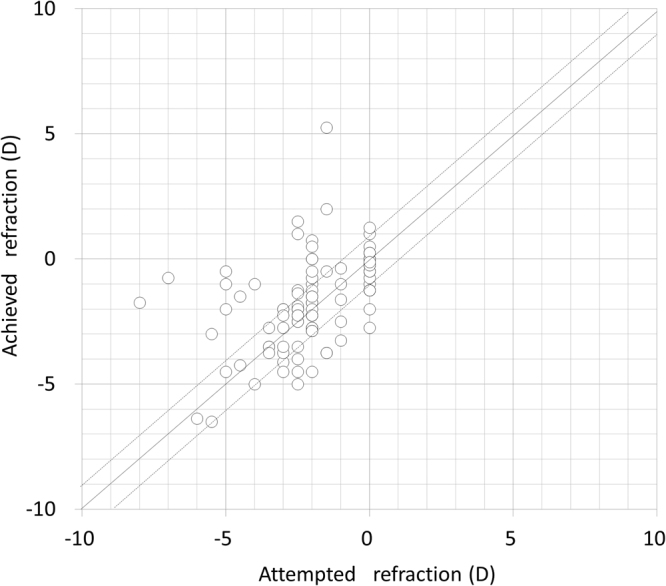
A scatter plot of the attempted versus the achieved refraction (manifest spherical equivalent) 1 month postoperatively in eyes with cataract and keratoconus. Thirty seven (36%) and 64 (63%) of 102 eyes were within ±0.5 D and 1.0 D, respectively, of the targeted correction.

**Figure 2:**
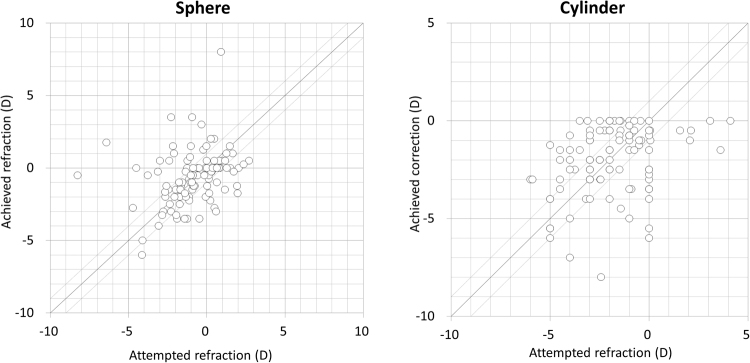
Scatter plots of the attempted versus the achieved refraction (sphere and cylinder) 1 month postoperatively in eyes with cataract and keratoconus.

**Figure 3:**
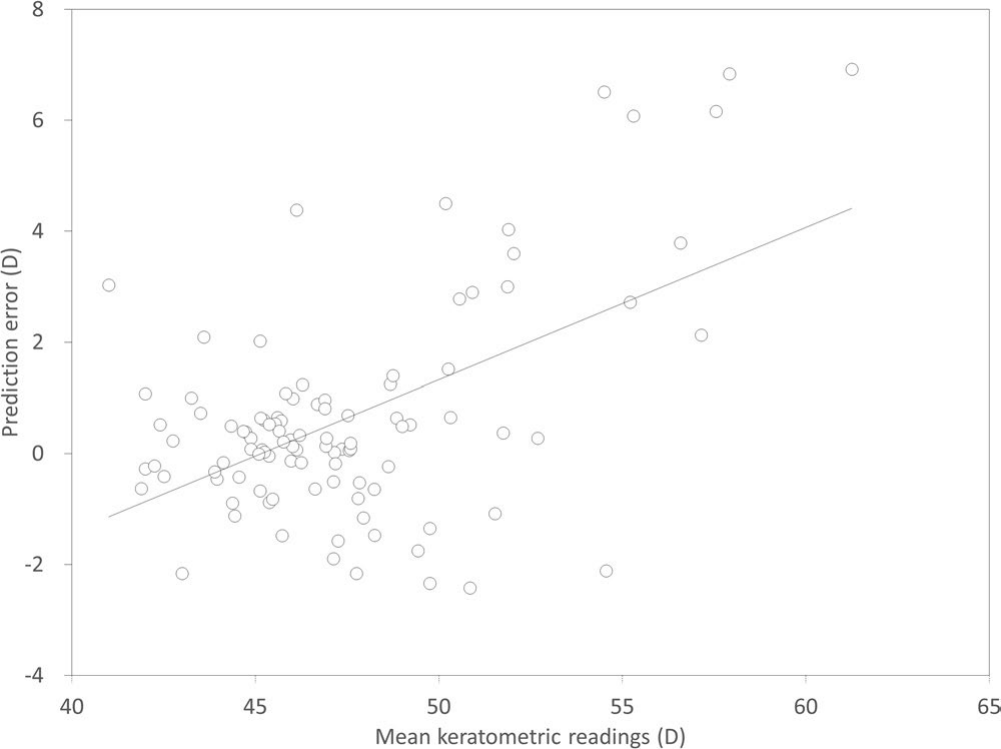
A graph showing a significant association between the prediction error and the keratometric values (Pearson correlation coefficient r =−0.545, p < 0.001).

**Figure 4:**
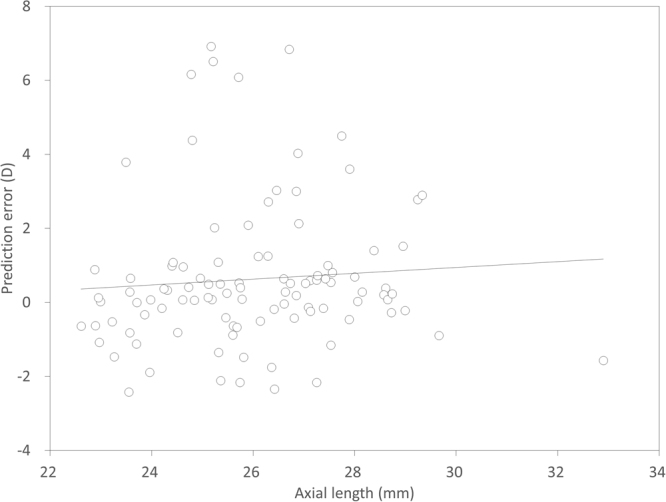
A graph showing no significant correlation between the prediction error and the axial length (Pearson correlation coefficient r = 0.163, p = 0.101).

Scatter plots of the attempted versus the achieved refraction (manifest spherical equivalent, sphere, and cylinder) 1 month postoperatively is shown in Figs 1 and 2. The achieved refraction of −1.75 (−2.75, −0.50) D was significantly more hyperopic than the targeted refraction of −2.28 (−2.89, −0.84) D (p = 0.001). Table 2 shows the prediction error, absolute error, and percentage of eyes within ±0.5 D and 1.0 D of the targeted correction, according to the stage of keratoconus. The IOL power prediction errors and the absolute errors are 0.27 (−0.42, 0.98) D and 0.65 (0.29, 1.56) D, respectively. Thirty seven (36%) and 64 (63%) of 102 eyes were within ±0.5 D and 1.0 D, respectively, of the targeted correction. In eyes with mild, moderate, and severe keratoconus, 56 (80%), 8 (32%), and 0 (0%) of 102 eyes were within ±1.0 D of the targeted correction, respectively. We found a significant correlation between the prediction error (spherical equivalent) and the keratometric values (Pearson correlation coefficient r = 0.545, p < 0.001) (Fig. 3), but no significant correlation between the prediction error and the axial length (r = 0.163, p = 0.101) (Fig. 4). We found a significant correlation of the keratometric values with the prediction spherical error (r = 0.400, p < 0.001), but not with the prediction cylindrical error (r =−0.023, p = 0.822).

**Table 2:** Predictability outcomes according to the grade of keratoconus.

Grade Mean keratometry	Low ≤ 48 D	Moderate > 48 D, ≤55 D	Severe > 55 D	P value
Number of eyes (%)	70 (69%)	25 (25%)	7 (7%)	
Prediction error	0.09 (−0.42, 0.58) D	0.52 (−1.08, −2.78) D	3.79 (2.90, 6.50) D	<0.001
Absolute error	0.52 (0.21, 0.89) D	1.47 (0.64, 2.78) D	3.79 (2.90, 6.50) D	<0.001
within ±0.5D (%)	33 (47%)	4 (16%)	0 (0%)	
within ±1.0D (%)	56 (80%)	8 (32%)	0 (0%)	

D = diopter.

For subgroup analysis, the total corneal refractive power was available for IOL power calculation in 51 (50%) eyes. Assuming that the total corneal refractive power was used for the keratometric readings, the achieved refraction of −1.50 (−2.25, −0.50) D was significantly more myopic than the targeted refraction of −1.45 (−2.26, 0.02) D (p = 0.013). The IOL power prediction errors and the absolute errors are −0.55 (−1.25, 0.20) D and 0.86 (0.34, 1.57) D, respectively. Eleven (22%) and 28 (55%) of 51 eyes were within ±0.5 D and 1.0 D, respectively, of the targeted correction. We found no significant correlation of the keratometric values with the prediction error (spherical equivalent) (r = 0.244, p = 0.084), or with the axial length (r = 0.112, p = 0.433). We found a significant correlation of the keratometric values with the prediction spherical error (r = 0.295, p = 0.036), but not with the prediction cylindrical error (r =−0.227, p = 0.108).

### Secondary Surgeries/Adverse Events

Neither cataract incision-related complication, keratectasia, significant IOL misalignment or rotation of the toric IOL, nor other vision-threatening complications was seen at any time during the observation period.

### Predictability with Other IOL Calculation Formulas

The IOL power prediction errors and the absolute errors using the SRK/T, Haigis, Holladay 1, Holladay 2, Hoffer Q, and SRK II formulas were summarized in Table 3. The variance in the prediction error was statistically significant (ANOVA, p = 0.004). We found significant differences in the prediction error determined by the SRK/T formula and by the Haigis formula (p = 0.027, Dunnett test), and by the SRK/T formula and by the Hoffer Q formula (p = 0.031). We also found significant differences in percentages of eyes within ±1.0 D determined by the SRK/T formula and by the Haigis formula (p = 0.005, Fisher’s exact test), and by the SRK/T formula and by the Hoffer Q formula (p = 0.024).

**Table 3:** Predictability outcomes using various intraocular lens power calculation formulas.

Formula	SRK/T	Haigis	Holladay 1	Holladay 2	Hoffer Q	SRK II	P value
Prediction error	0.27 (−0.42, 0.98) D	1.22 (0.47, 2.22) D	0.76 (0.23, 1.91) D	0.97 (0.46, 1.65) D	1.08 (0.46, 2.29) D	−0.45 (−1.39, 0.62) D	0.003
Absolute error	0.65 (0.29, 1.56) D	1.31 (0.63, 2.29) D	0.96 (0.48, 2.03) D	1.02 (0.53, 1.66) D	1.15 (0.60, 2.29) D	1.04 (0.51, 1.95) D	0.062
within ±0.5D	36%	22%	27%	27%	21%	25%	
within ±1.0D	63%	42%	51%	51%	46%	49%	

D = diopter.

## Discussion

Our multicenter study showed that phacoemulsification with IOL implantation was good in measures of safety and efficacy in cataractous patients with keratoconus, and that the predictability was also good in mild keratoconus, but not in moderate to severe keratoconus. Based on our clinical findings, it should be emphasized that a large amount of hyperopic shift occurred in advanced keratoconus in the current study. As far as we can ascertain, this is the largest study to investigate the clinical outcomes of contemporary cataract surgery for such patients. This is also the first multicenter study to focus on the refractive accuracy of cataract surgery, and to assess it using the total corneal refractive power for keratoconic patients. We estimated the magnitude of corneal refractive power on an approximately 2.4-mm ring using the standardized ratio of anterior and posterior corneal power (1.3375), since this measurement was simple and easy to perform in daily practice. Although the change in the posterior corneal surface plays more subtle role in optical performance than that in the anterior corneal surface, because of the smaller change in the refractive index, the adoption of total corneal refractive power may be helpful to accurately calculate the IOL power especially for advanced keratoconus. Our study also showed that a slight, but significant, myopic shift occurred, when the total corneal refractive power was used for keratometry. We believe that this information will be helpful for determining the accurate IOL power for keratoconus in daily practice.

Previous studies on the clinical outcomes of non-toric IOL implantation for keratoconus were summarized in Table 4. Leccisotti *et al*. showed that the safety and efficacy indices were 1.38 and 0.87 after refractive lens exchange, respectively, but that the IOL exchange due to inaccurate power occurred in 32% of eyes^1^. Thebpatiphat *et al*. stated, in a preliminary study on 9 keratoconic patients, that patients with mild keratoconus had a mean postoperative refraction of −1.44 ± 1.69 (mean ± standard deviation) D, which was significantly lower than those with moderate keratoconus, who had a mean postoperative refraction of −5.85 ± 3.94 D^2^. Watson *et al*. demonstrated that it is a suitable option for spherical IOL selection for eyes with a mean keratometry of 55 D or more to use the actual keratometric values with a target of low myopia, but that the use of actual keratometric values can result in a large hyperopic error for severe keratoconus^3^. Park *et al*. described that hyperopic shift was noted because localized posterior elevation of the cornea is not considered in the conventional IOL power calculation in a patient having posterior keratoconus^11^. The latter two findings were in agreement with our findings in the current study. We should be aware that hyperopic shift may occur after cataract surgery, especially in eyes with severe keratoconus or posterior keratoconus. Toric IOL implantation has been also shown to be a feasible option for cataractous eyes having mild non-progressive keratoconus^4–10^. Previous studies on the clinical outcomes of toric IOL implantation for keratoconus were also summarized in Table 4. Although toric IOL implantation is usually applied for mild non-progressive keratoconus, it should be noted that a hyperopic shift can occur even in such patients, since these patients tend to make a high demand for uncorrected visual acuity after toric IOL implantation. In cases of progressive keratoconus, corneal collagen cross-linking (CXL) is known to be effective for halting the disease^12^. The two-step procedures of CXL followed by phacoemulsification with toric IOL implantation may be one of viable surgical options for such cataractous eyes with progressive keratoconus^13^.

**Table 4:** Summary of previous studies on non-toric and toric intraocular lens implantation for keratoconus.

Author	Number of eyes	Age (years)	Follow-up (months)	IOL type	IOL calculation formula	Keratometry	UDVA	CDVA	Spherical equivalent	Astigmatism	within ± 0.5 D(%)	within ± 1.0 D(%)
Leccisotti *et al*.^1^	34	56.7 ± 10.4	17.4 ± 5.1	non-toric	Holladay 2	topography	0.48 ± 0.25 (decimal)	0.76 ± 0.23 (decimal)	−1.31 ± 1.08	1.22 ± 1.37	9	47
Thebpatiphat *et al*.^2^	12	55.3 ± 11.8	3	non-toric	SRKI, SRKII, SRK-T	keratometer topography	0.63 ± 0.47	0.21 ± 0.13	−1.44 ± 1.69 (mild), −5.85 ± 3.94 (moderate)	N.A.	N.A.	N.A.
Watson *et al*.^3^	91	59	33	non-toric	SRK-T	keratometer	N.A.	0.3 (mild) 0.2 (moderate) 0.2 (severe)	−1.0 (mild)-1.5 (moderate) −5.4 (severe)	N.A.	N.A.	60 (mild) 41.9 (moderate) N.A. (severe)
Navas *et al*.^4^	2	55, 46	12	toric	SRKII	topography	20/25	N.A.	−0.5, 0	0.5	N.A.	N.A.
Visser *et al*.^5^	3	78, 64, 64	6	toric	SRK-T	keratometer	20/50, 20/130, 20/30	20/30, 20/40, 20/25	−0.75, −3.25, −0.63	1.5, 1.5, 0.75	N.A.	N.A.
Jaimes *et al*.^6^	19	48.2 ± 6.6	7.9 ± 6.6	toric	SRKII	topography	0.29 ± 0.23	0.11 ± 0.12	−0.46 ± 1.12	1.36 ± 1.17	38	85
Nanavaty *et al*.^7^	12	63.4 ± 3.5	9.0 ± 8.1	toric	company proprietary software	N.A.	20/40	20/30	0.10 ± 0.60	0.60 ± 1.10	N.A.	N.A.
Alió *et al*.^8^	17	56.6 ± 12.5	9.1	toric	Hoffer Q, SRK-T	keratometer	0.32 ± 0.38	0.20 ± 0.36	−0.62 ± 0.97	1.40 ± 1.13	71	71
Hashemi *et al*.^9^	23	59 ± 12.8	3	toric	Hoffer Q (axial length < 22 mm) SRK II (22 to 24.5 mm) Holladay I (24.5 to 26 mm) SRK/T (> 26 mm)	keratometer topography	0.27 ± 0.18 (mild) 0.34 ± 0.19 (moderate) 0.38 ± 0.29 (severe)	0.16 ± 0.09 (mild) 0.18 ± 0.12 (moderate) 0.35 ± 0.13 (severe)	−0.58 ± 0.95 (mild) −0.34 ± 0.90 (moderate) 0.50 ± 0.58 (severe)	1.83 ± 0.90 (mild) 1.25 ± 0.96 (moderate) 4.67 ± 2.31 (severe)	N.A.	N.A.
Kamiya *et al*.^10^	19	63.1 ± 9.1	3	toric	SRK-T	keratometer	0.46 ± 0.33	−0.01 ± 0.09	N.A.	0.70 ± 0.60	68	95

IOL = intraocular lens, UDVA = uncorrected visual acuity, CDVA = corrected visual acuity, D = diopter, N.A. = not available.

Results was expressed as mean ± standard deviation.

It is still challenging to precisely determine the IOL power for maximizing the postoperative visual performance and subsequent patient satisfaction in keratoconic patients. In the current study, the prediction error by the use of the SRK/T formula was significantly better than that by the use of the Haigis formula or the Hoffer Q formula. Leccisotti *et al*. reported that the SRK II formula provided more accurate IOL power than SRK I and SRK/T formulas in patients with mild keratoconus. Their findings were not in accordance with our results. However, they only included 5 eyes with mild keratoconus, and thus statistical analysis cannot be performed in their study^1^. Hashemi *et al*. showed that the lowest mean absolute error was seen with corneal topography-derived keratometry using the SRK/T formula for mild to moderate keratoconus, and with corneal topography-derived keratometry and manual keratometry using the SRK/T and SRK II formulas for severe keratoconus^9^. Although we accept that that it is still difficult to accurately select the IOL power for severe keratoconus, even when the SRK/T formula was used its calculation, we believe that it will be helpful for selecting the proper IOL power calculation formula in a clinical setting.

This study is burdened with several limitations. Firstly, this study was conducted in a retrospective fashion. A randomized, controlled study may provide further information for confirming the authenticity of these results. Secondly, we did not measure corneal refractive power of the posterior surface or corneal higher-order aberrations in this study. Thirdly, several kinds of IOLs were used by multiple surgeons in the current study, since the preferred IOLs were different among these 4 institutions. Although various constants were optimized in each institution in this study, it would be ideal for using the same IOL implanted by a single surgeon to clarify this point. Fourthly, the target refraction was set at emmetropia, slight myopia, and similar refraction in the fellow eye, based on preoperative refraction and patient preference for vision. Fifthly, the follow-up period was up to 1 month postoperatively. Although the predictability was evaluated at 1 month postoperatively in most studies of modern cataract surgery, a further long-term observation may be necessary.

In conclusion, our multicenter study supports the view that phacoemulsification with IOL implantation was safe and effective, and that the accuracy was also good in mild keratoconus, but not in moderate to severe keratoconus. We should be aware that a large amount of hyperopic shift occurred in advanced keratoconic patients, when the keratometric readings were used for the IOL power calculation, and that a slight, but significant, myopic shift occurred, when total corneal refractive power was used. We believe that this information is helpful for determining the accurate IOL power in keratoconic patients for keratoconus in daily practice.

## Methods

### Study Population

The protocol was registered with the University Hospital Medical Information Network Clinical Trial Registry (000018425). A total of one hundred two eyes of the 71 keratoconic patients (33 men and 38 women) who had undergone standard phacoemulsification with IOL implantation (Kitasato University Hospital, Miyata Eye Hospital, Tokyo Dental College Ichikawa Hospital, and Osaka University Hospital) in Japan from January 2008 to December 2016, and who completed at least a 1-month follow-up, were included in this cohort study. The patients were recruited in a continuous cohort. Keratoconus was diagnosed by four experienced clinicians (K.K., K.M., J.S., and N.M.) based on evident findings characteristic of keratoconus (e.g., corneal topography with asymmetric bow-tie pattern with or without skewed axes), and at least one keratoconus sign (e.g., stromal thinning, conical protrusion of the cornea at the apex, Fleischer ring, Vogt striae, or anterior stromal scar) on slit-lamp examination^14^. The severity of keratoconus was classified as mild, moderate, or severe, according to the average keratometric readings and based on the Amsler Krumeich classification^15^. The study population were divided into 3 subgroups: mild keratoconus was defined as average keratometric readings of ≤48 diopters (D), moderate keratoconus as >48 D and ≤55 D, and severe keratoconus as >55 D^3,15^. The patients who wore rigid gas permeable and soft contact lenses were asked to stop using them for 3 and 2 weeks, respectively, prior to biometry. Using the envelope technique, we randomly included one eye per patient for statistical analysis to control the simultaneous effects of both eyes. This sample size offered 91.3% statistical power at the 5% level to detect a 0.20-difference in logarithm of the minimal angle of resolution (logMAR) of visual acuity, when the standard deviation of the mean difference was 0.50. The exclusion criteria was as follows: postoperative spectacle corrected distance visual acuity (CDVA) of >0.32 logMAR (equivalent to decimal visual acuity of <0.5) (because of unreliable refraction), pellucid marginal degeneration having inferior corneal thinning with ectasia above the area of thinning, any history of ocular surgery, ocular trauma, or other concomitant eye diseases. Patient data was anonymized before access and/or analysis. Written informed consent was obtained from all patients for the surgery after explanation of the nature and possible consequences of the study. This retrospective review of the data was approved by the Institutional Review Board at Kitasato University (B15–144) and followed the tenets of the Declaration of Helsinki. Our Institutional Review Board waived the requirement for informed consent for this retrospective study.

### Surgical Procedures

For cataract surgery, standard phacoemulsification was performed by experienced surgeons (K.K., K.M., J.S., and N.M.). The surgical technique consisted of a capsulorhexis, nucleus and cortex extraction, and a monofocal IOL implantation. We selected toric IOL only for mild non-progressive keratoconus with corneal astigmatism of ≥1.5 D, low higher-order aberrations, and contact lens intolerance^10^. A web-based toric IOL calculator program was used to determine the optimal cylinder power and alignment axis of the IOL. In the remaining eyes, we used several non-toric IOLs based on the surgeons’ preference in each institution. In 27, 72 and 3 of 102 eyes, we selected emmetropia, slight myopia for near monovision, similar refraction in the fellow eye, respectively, as the target refraction. Postoperatively, steroidal, antibiotic, and bromfenac sodium medications were topically administered for 1 month, the dose being reduced gradually thereafter.

### Assessment of Prediction Error and Absolute Error

IOL power calculations were performed by the SRK/T formula using the axial length and the keratometric readings measured by a partial coherence interferometer (IOL Master 500^TM^, Carl Zeiss Meditec, Jena, Germany) without any correction. For subgroup analysis, we also used the total corneal refractive power measured by a rotating Scheimpflug imaging instrument (Pentacam HR^TM^, version 1.20, Oculus, Wetzlar, Germany) on the central 15° ring (equal to the 3.0-mm ring) for IOL power calculations. The optimized A-constants were used in each institution. Each measurement was repeated at least 3 times and the mean value was used for the analysis. The prediction errors defined by subtracting the predicted postoperative refraction from the postoperative spherical equivalent 1 month postoperatively, these absolute values, and the percentages of the prediction errors within ±0.5 D and ±1.0 D were calculated.

In order to determine which formulas provide the most accurate IOL power, we retrospectively calculated the prediction and absolute errors assuming that the Haigis, Holladay 1, Holladay 2, Hoffer Q, and SRK II formulas were used. For this analysis, we also used the anterior chamber depth (ACD) measured by the same partial coherence interferometer. The optimized constants were also used in each institution.

### Statistical Analysis

All statistical analyses and statistical power calculation were performed using a commercially available statistical software (BellCurve for Excel, Social Survey Research Information Co, Ltd., Tokyo, Japan). The normality of all data samples was first checked by the Kolmogorov-Smirnov test. Since the data fulfilled the criteria for normal distribution, the Student t test was used for statistical analysis to compare the pre- and post-surgical data, and the Pearson correlation coefficient was used to assess the relationship between the two variables. One-way analysis of variance (ANOVA) was used for the analysis of the prediction errors using several IOL power calculation formulas, the Dunnett test being employed for multiple comparisons. The Fisher’s exact test was used to compare the percentages of eyes within ± 1.0 D of the targeted correction. Unless otherwise indicated, the results are expressed as the median (25th and 75th percentile), a value of p < 0.05 was considered statistically significant.

### Data availability

The datasets generated during and/or analysed during the current study are available from the corresponding author on reasonable request.

## References

[1] Leccisotti A (2006). Refractive lens exchange in keratoconus. J Cataract Refract Surg..

[2] Thebpatiphat N, Hammersmith KM, Rapuano CJ, Ayres BD, Cohen EJ (2007). Cataract surgery in keratoconus. Eye Contact Lens..

[3] Watson MP (2014). Cataract surgery outcome in eyes with keratoconus. Br J Ophthalmol..

[4] Navas A, Suárez R (2009). One-year follow-up of toric intraocular lens implantation in forme fruste keratoconus. J Cataract Refract Surg..

[5] Visser N, Gast ST, Bauer NJ, Nuijts RM (2011). Cataract surgery with toric intraocular lens implantation in keratoconus: a case report. Cornea..

[6] Jaimes M (2011). Refractive lens exchange with toric intraocular lenses in keratoconus. J Refract Surg..

[7] Nanavaty MA, Lake DB, Daya SM (2012). Outcomes of pseudophakic toric intraocular lens implantation in keratoconic eyes with cataract. J Refract Surg..

[8] Alió JL (2014). MICS with toric intraocular lenses in keratoconus: outcomes and predictability analysis of postoperative refraction. Br J Ophthalmol..

[9] Hashemi H, Heidarian S, Seyedian MA, Yekta A, Khabazkhoob M (2015). Evaluation of the results of using toric IOL in the cataract surgery of keratoconus patients. Eye Contact Lens..

[10] Kamiya K, Shimizu K, Miyake T (2016). Changes in astigmatism and corneal higher-order aberrations after phacoemulsification with toric intraocular lens implantation for mild keratoconus with cataract. Jpn J Ophthalmol..

[11] Park DY, Lim DH, Chung TY, Chung ES (2013). Intraocular lens power calculations in a patient with posterior keratoconus. Cornea..

[12] Wollensak G, Spoerl E, Seiler T (2003). Riboflavin/ultraviolet-a-induced collage crosslinking for the treatment of keratoconus. Am J Ophthalmol..

[13] Abou Samra, W. A., Awad, E. A., & El Kannishy, A. H. Objective and subjective Outcome of clear lensectomy with toric IOL implantation after corneal collagen cross-linking in selected cases of keratoconus. *Eye Contact Lens*. 2016 Oct 3. [Epub ahead of print]. 10.1097/ICL.0000000000000333 27749500

[14] Rabinowitz YS (1998). Keratoconus. Surv Ophthalmol..

[15] Krumeich JH, Kezirian GM (2009). Circular keratotomy to reduce astigmatism and improve vision in stage I and II keratoconus. J Refract Surg..

